# Reduced serum magnesium is associated with the occurrence of diabetic macular edema in patients with diabetic retinopathy: A retrospective study

**DOI:** 10.3389/fmed.2022.923282

**Published:** 2022-09-20

**Authors:** Xiaoli Xiang, Zijia Ji, Tingwang Jiang, Zhengru Huang, Jing Yan

**Affiliations:** ^1^Department of Ophthalmology, The Affiliated Changshu Hospital of Xuzhou Medical University, Changshu, China; ^2^Department of Ultrasonography, The Affiliated Changshu Hospital of Xuzhou Medical University, Changshu, China; ^3^Department of Key Laboratory, The Affiliated Changshu Hospital of Xuzhou Medical University, Changshu, China; ^4^School of Health Service Management, Anhui Medical University, Hefei, China

**Keywords:** magnesium, diabetic macular edema, diabetic retinopathy, diabetes mellitus, vision loss

## Abstract

Serum magnesium levels have been reported to reflect the risk of diabetic retinopathy (DR); however, the effect of serum magnesium level on diabetic macular edema (DME) remains unclear. Here, we investigated the association between the serum magnesium levels and DME in patients with DR. Patients with DR were recruited between January 2018 and June 2021. A total of 519 such patients were included in this study. All patients underwent a standardized clinical ophthalmic examination by an experienced ophthalmologist, and an assay was conducted to determine the serum magnesium concentration. Compared with the non-DME group, the DME group had a higher proportion of insulin use and a higher level of serum ischemia-modified albumin and fasting plasma glucose. The serum magnesium and calcium levels were lower in the DME group than in the non-DME group (*P* < 0.05). Higher magnesium levels were negatively associated with DME after adjustment for relevant covariates. Compared with the participants in the lowest magnesium quartile, those in the fourth quartile showed a significantly lower risk of DME after adjustment [odds ratio (OR), 0.294; 95% confidence interval, 0.153–0.566; *P* < 0.0001]. Considering the potentially different effects of serum magnesium on the development of DME in patients with DR based on age, DR staging and insulin use, stratified analysis was performed by considering these factors. Among insulin-using patients with non-proliferative DR who were < 66 years of age, those in the third and fourth quartile of serum magnesium were less likely to develop DME than those in the lowest quartile of serum magnesium [OR (95% CI), 0.095 (0.014–0.620), 0.057 (0.011–0.305); *P* = 0.014, 0.001]. Overall, a higher serum magnesium level was associated with a lower risk of DME in patients with DR. Furthermore, patients with DR who used insulin were more likely to develop DME. Long-term studies on oral magnesium supplements are needed to determine whether maintaining the serum magnesium levels in a higher physiological range can reduce the risk of DME in patients with DR.

## Introduction

Diabetes is a global health problem with high incidence (1) and can lead to several complications. Diabetic retinopathy (DR) is a unique diabetic microvascular complication that is the main cause of adult vision loss in many countries (2). Diabetic macular edema (DME), a buildup of fluid in the central retina, can occur at various stages of DR. DME is the main cause of central vision loss in patients with diabetes and can affect activities of daily living, such as reading and driving, and severely decrease their quality of life (3). Increased retinal vascular permeability, followed by edema and hard exudate, are the main clinical features (4). The prevalence of DR and DME in patients with diabetes has been reported to be 35.4 and 7.4%, respectively (5).

Serum magnesium levels have been reported to reflect the risk of DR (6). Magnesium is the second largest intracellular cation in the human body, after potassium. It is a cofactor of more than 600 enzymes and an activator of more than 200 enzymes (7). Moreover, magnesium plays a role in many metabolic pathways, including glycolysis, β-oxidation, and insulin signal transduction (8, 9). However, the effect of serum magnesium level on DME remains unclear. Therefore, the purpose of this study was to determine the clinical value of serum magnesium levels in the diagnosis of DME by analyzing the relationship between the serum magnesium levels and DME in patients with DR.

## Materials and methods

### Study population

This was a single-center retrospective study. A total of 519 patients with type 2 diabetes diagnosed with DR using color fundus imaging or fluorescein angiography who were admitted to the Department of Ophthalmology of the Affiliated Changshu Hospital of Xuzhou Medical University between January 2018 and June 2021 were enrolled in this study.

The exclusion criteria were as follows: (1) a history of eye trauma and eye surgery; (2) macular degeneration, macular anterior membrane, vitreomacular traction syndrome, and other fundus diseases; (3) pregnancy; (4) systemic diseases (neurological diseases; tumors; infections; severe hepatic, cardiac, and renal insufficiency; history of malignant tumors; history of hematology; history of autoimmune diseases; history of malabsorption; history of chronic diarrhea; and history of chronic kidney disease); (5) neuropsychiatric disorders leading to inability to cooperate with the examination; and (6) intake of thiazide diuretics, aminoglycoside antibiotics, amphotericin B, and other drugs that may affect serum magnesium excretion.

This study was approved by the Ethics Committee of the Affiliated Changshu Hospital of Xuzhou Medical University (Changshu, China; approval number: 2019082). All patients provided written informed consent upon admission.

### Baseline data collection

Clinical data, including age, sex, history of hypertension, systolic blood pressure, diastolic blood pressure, and use of hypoglycemic drugs, were collected. The height and weight of all patients were measured while being in the standing position at admission. The body mass index was calculated by dividing the weight by the height squared (kg/m^2^). Blood pressure was read as the patient’s sitting blood pressure measured in a quiet state by a professional nurse at admission. Fasting (> 8 h) blood samples were collected for analysis. Blood (cell) count and serum biochemical variables (i.e., urea, uric acid, calcium, magnesium, ischemia modified albumin, retinol binding protein, fasting blood glucose) were assessed using a conventional automated blood analyzer (AU5800 Series Chemistry Analyzers; Beckman Coulter, Brea, CA, USA).

### Definition of diabetic macular edema and ophthalmic examination

Each patient underwent a standardized clinical ophthalmic examination by an experienced fundus surgeon, including a review of ophthalmic history, measurement of visual acuity, and intraocular pressure, slit-lamp examination, and dilated fundus examination under a slit lamp. Color fundus imaging and optical coherence tomography were performed after pupil dilation, and fluorescein angiography was performed, where necessary. The diagnostic criteria for DR were based on the International Clinical Diabetic Retinopathy Disease Severity Scale (10). DME was defined according to the Early Treatment Diabetic Retinopathy Study report as any retinal thickening or hard exudate within the diameter of a disc in the fovea in the presence of DR features (11). Macular edema was confirmed by optical coherence tomography (Cirrus HD-OCT5000; Carl Zeiss, Jena, Germany) or fluorescein angiography (Spectralis HRA; Heidelberg, Germany).

### Statistical analysis

The measurement data were normally distributed, represented by mean ± standard deviation values, and comparisons between the groups were made using the independent-sample *t*-test. The measurement data were skewness distribution data, represented by quartiles, and the Mann–Whitney *U*-test was used for intergroup comparison. Categorical variables are expressed as frequency (percentage) and were compared between groups using the χ^2^-test. Spearman’s rank correlation was used to analyze the correlation between serum magnesium levels and other clinical variables. A strong correlation was defined as a correlation coefficient > 0.4 and a *P*-value < 0.01. Binary logistic regression models were used to examine the relationship between serum magnesium levels and DME outcome. Variables with a *P*-value < 0.1 in univariate logistic regression were included in the multivariate logistic regression model. Statistical analyses were performed using SPSS 19.0 statistical software (IBM Corp., Armonk, NY, USA). All tests were two-sided, and statistical significance was set at *P* < 0.05.

## Results

### Baseline characteristics

In total, 519 patients with DR participated in this study. The patients in the DME group were older than those in the non-DME group. Although the proportion of men in the DME group was higher than that in the non-DME group, the proportion of women was still higher than that of men. Compared to the non-DME group, the DME group had a higher proportion of insulin use to control blood glucose and a higher level of serum ischemia-modified albumin and fasting plasma glucose. The serum magnesium and calcium levels were lower in the DME than in the non-DME group (*P* < 0.05). The clinical characteristics of all patients are listed in Table 1.

**TABLE 1:** Comparison of clinical characteristics between the diabetic macular edema (DME) and non-DME groups.

Variable	Non-DME (*n* = 296)	DME (*n* = 223)	*P*-value
Age (years)	61.17 ± 9.22	69.85 ± 8.46	<0.0001*
Male, *n* (%)	102 (45.7%)	144 (48.6%)	<0.0001*
PDR, *n* (%)	15 (15.1%)	104 (46.6%)	<0.0001*
BMI (kg/m^2^)	23.82 ± 2.85	23.98 ± 2.99	0.620
Hypertension, *n* (%)	233 (78.7%)	181 (81.2%)	0.492
SBP (mmHg)	146.26 ± 18.56	145.16 ± 19.97	0.243
DBP (mmHg)	82.87 ± 10.71	82.01 ± 10.55	0.249
Insulin, *n* (%)	89 (30.1%)	146 (65.5%)	<0.0001*
**Laboratory findings**			
Urea (mmol/L)	6.96 ± 2.97	7.35 ± 3.18	0.136
UA (μmol/L)	348.77 ± 114.05	345.10 ± 113.13	0.586
IMA (U/mL)	57.15 ± 14.23	58.82 ± 13.25	0.005*
RBP (μg/mL)	50.06 ± 17.65	48.92 ± 22.00	0.324
Calcium (mmol/L)	2.32 ± 0.12	2.30 ± 0.13	0.008*
Magnesium (mmol/L)	0.86 ± 0.14	0.79 ± 0.19	<0.0001*
FPG (mmol/L)	6.88 ± 2.02	7.09 ± 2.64	0.019*
Neutrophil (◊10^9^/L)	3.79 ± 1.40	3.89 ± 1.43	0.673
Lymphocyte (◊10^9^/L)	1.49 ± 0.56	1.47 ± 0.52	0.651
Blood platelet (◊10^9^/L)	171.00 ± 54.59	171.58 ± 54.49	0.466

DME, diabetic macular edema; PDR, proliferative diabetic retinopathy; BMI, body mass index; SBP, systolic blood pressure; DBP, diastolic blood pressure; UA, uric acid; IMA, ischemia modified albumin; RBP, retinol binding protein; FPG, fasting plasma glucose.

*P < 0.05, significant difference.

### Frequency of diabetic macular edema among patients with different serum magnesium levels

**FIGURE 1:**
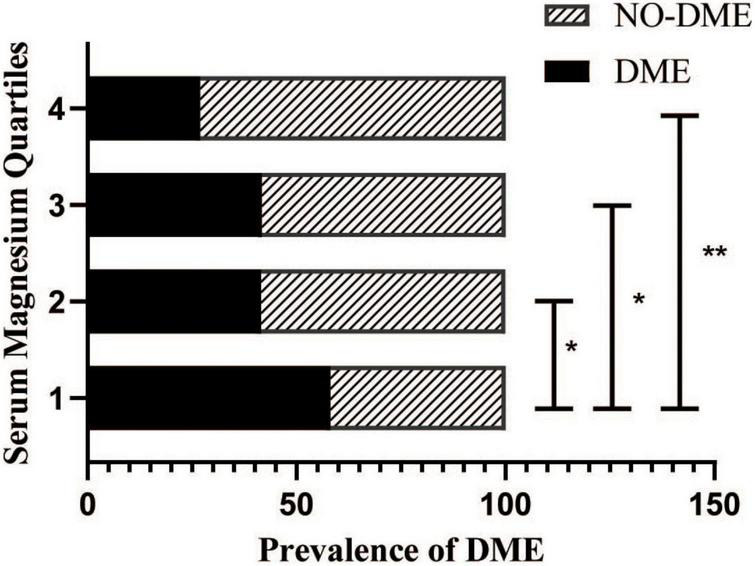
Prevalence of diabetic macular edema (DME) in patients with diabetic retinopathy stratified by the serum magnesium levels.

The serum magnesium levels of patients were divided into four groups according to the following quartiles: Q1 (≤ 0.78 mmol/L), Q2 (0.78–0.85 mmol/L), Q3 (0.85–0.91 mmol/L), and Q4 (> 0.91 mmol/L), respectively. The incidence of DME in all groups (58.1, 41.5, 41.7, and 26.9%, respectively) decreased with increasing serum magnesium levels. Compared with Q1, groups Q2, Q3, and Q4 showed statistically significant differences (*P* = 0.0051, *P* = 0.0121, *P* < 0.0001, respectively) (Figure 1). There was also a difference in the incidence of DME between the Q2, Q3, and Q4 groups (*P* = 0.0180, *P* < 0.0001, *P* = 0.0246, respectively).

### Comparison of risk for diabetic macular edema in patients with different serum magnesium levels

Table 2 shows the results from the univariate binary logistic regression model used to analyze the factors influencing DME, including serum magnesium, serum calcium, age, DR staging, and insulin use, in the multiple binary logistic regression model (Table 3). Multiple regression analysis (Table 3) showed that the serum magnesium levels in Q2 [odds ratio (OR), 0.512; 95% confidence interval (CI), 0.322–0.815], Q3 (OR, 0.515; 95% CI, 0.313–0.848), and Q4 (OR, 0.266; 95% CI, 0.153–0.461) were negatively correlated with the risk of DME compared with the serum magnesium levels in group Q1.

**TABLE 2:** Univariate analysis of factors influencing DME.

Variable	Univariate analysis	
	OR (95% CI)	*P*-value
Age	0.897 (0.876–0.918)	<0.0001*
Sex	0.890 (0.628–1.261)	0.511
DR staging	16.372 (9.146–29.306)	<0.0001*
BMI	0.981 (0.924–1.041)	0.523
Hypertension	1.165 (0.753–1.802)	0.492
SBP	1.003 (0.994–1.012)	0.521
DBP	1.008 (0.991–1.024)	0.360
Insulin	4.410 (3.042–6.393)	<0.0001*
**Laboratory findings**		
Urea	1.043 (0.985–1.105)	0.149
UA	1.000 (0.998–1.001)	0.723
IMA	1.008 (0.994–1.022)	0.247
RBP	0.996 (0.987–1.005)	0.369
Calcium	0.221 (0.057–0.859)	0.029*
Magnesium	0.043 (0.010–0.198)	<0.0001*
FPG	1.040 (0.964–1.121)	0.310
Neutrophil	1.050 (0.929–1.187)	0.434
Lymphocyte	0.939 (0.682–1.292)	0.698
Blood platelet	1.000 (0.997–1.003)	0.905

DME, diabetic macular edema; OR, odds ratio; CI, confidence interval; DR, diabetic retinopathy; BMI, body mass index; SBP, systolic blood pressure; DBP, diastolic blood pressure; UA, uric acid; IMA, ischemia modified albumin; RBP, retinol binding protein; FPG, fasting plasma glucose.

*P < 0.05, significant difference.

**TABLE 3:** Multivariate analysis of factors influencing DME.

Quartiles	Model 1		Model 2		Model 3		Model 4		Model 5	
	OR (95% CI)	*P*-value	OR (95% CI)	*P*-value	OR (95% CI)	*P*-value	OR (95% CI)	*P*-value	OR (95% CI)	*P*-value
Q1 (≤ 0.78)		<0.0001*		<0.0001*		<0.0001*		0.002*		0.012*
Q2 (0.78–0.85)	0.512 (0.322–0.815)	0.005*	0.526 (0.314–0.882)	0.015*	0.600 (0.349–1.030)	0.013	0.590 (0.343–1.016)	0.057	0.709 (0.396–1.269)	0.247
Q3 (0.85–0.91)	0.515 (0.313–0.848)	0.009*	0.621 (0.358–1.077)	0.090	0.824 (0.463–1.469)	0.104	0.828 (0.464–1.479)	0.523	0.767 (0.410–1.432)	0.405
Q4 (> 0.91)	0.266 (0.153–0.461)	<0.0001*	0.267 (0.144–0.494)	<0.0001*	0.295 (0.154–0.568)	<0.0001*	0.294 (0.153–0.566)	<0.0001*	0.310 (0.153–0.629)	0.001*
Age			0.897 (0.875–0.918)	<0.0001*	0.903 (0.881–0.925)	<0.0001*	0.902 (0.881–0.925)	<0.0001*	0.918 (0.894–0.941)	<0.0001*
Calcium					0.203 (0.045–0.927)	0.040	0.343 (0.072–1.647)	0.182	0.277 (0.052–1.464)	0.131
Insulin							3.449 (2.265–5.252)	<0.0001*	2.643 (1.683–4.152)	<0.0001*
DR staging									9.308 (4.913–17.635)	<0.0001*

DME, diabetic macular edema; OR, odds ratio; CI, confidence interval; DR, diabetic retinopathy. Logistic regression: Model 1, unadjusted for confounding variables; Model 2, adjusted for age; Model 3, adjusted for age and calcium; Model 4, adjusted for age, calcium, and insulin; Model 5, adjusted for age, calcium, insulin, and DR staging.

*P < 0.05, significant difference.

After adjusting for age, DR staging, and insulin use (Model 5), the negative correlation between serum magnesium and DME risk in the Q4 group was still statistically significant (Table 3). Higher serum magnesium levels were associated with a reduced risk of DME in patients with DR (OR, 0.310; 95% CI, 0.153–0.629). Insulin use (OR, 2.643; 95% CI, 1.683–4.152) and DR staging (OR, 9.308; 95% CI, 4.913–17.635) were associated with an increased risk of DME in patients with DR, whereas age was associated with a reduced risk of DME in patients with DR (OR, 0.918; 95% CI, 0.894–0.941).

### Subgroup analyses based on age, diabetic retinopathy staging, and insulin

Considering the potentially different effects of serum magnesium on the development of DME in patients with DR based on age, DR staging, and insulin use, stratified analysis was performed based on these factors. Among insulin-using patients with non-proliferative DR who were < 66 years of age, those in the third and fourth quartile of serum magnesium were less likely to develop DME than those in the lowest quartile of serum magnesium [OR (95% CI), 0.095 (0.014–0.620) and 0.057 (0.011–0.305), respectively; *P* = 0.014 and 0.001, respectively].

### The serum magnesium level prediction value for diabetic macular edema

**FIGURE 2:**
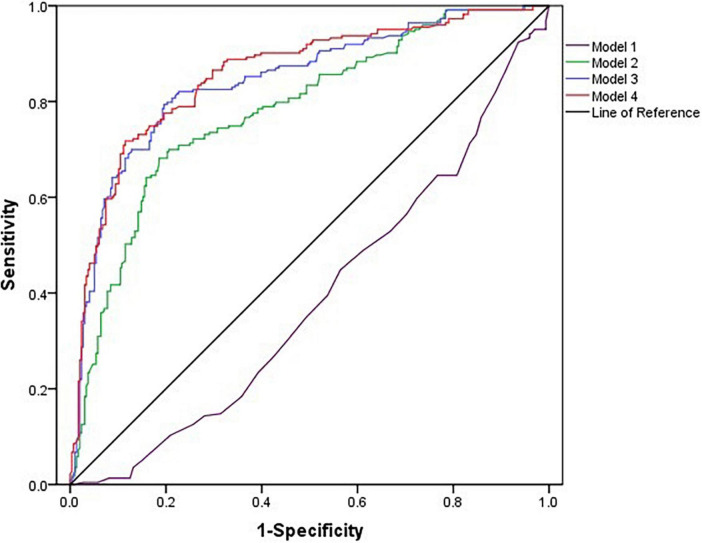
Receiver operating characteristic curves for diabetic macular edema.

As shown in Figure 2, marked Model 4 had the largest area under the curve (0.856; 95% CI, 0.823–0.890; Table 4) and a greater risk prediction value.

**TABLE 4:** The predictive value of serum magnesium alone and combined with age, DR staging, and insulin for the occurrence of DME.

Variables	AUC (95% CI)	Youden index	Sensitivity	Specificity	*P*-value
Model 1*	0.388 (0.340–0.436)	–0.012	0.924	0.064	<0.0001
Model 2*	0.780 (0.740–0.820)	0.496	0.709	0.760	<0.0001
Model 3*	0.844 (0.808–0.879)	0.598	0.794	0.804	<0.0001
Model 4*	0.856 (0.823–0.890)	0.603	0.717	0.885	<0.0001

DME, diabetic macular edema; DR, diabetic retinopathy; AUC, area under the curve; CI, confidence interval. Model 1, unadjusted; Model 2, Model 1 + age; Model 3, Model 2 + DR Staging; Model 4, Model 3 + insulin.

*P < 0.05, significant difference.

### Clinical parameters correlated with serum magnesium levels

The serum magnesium levels were positively correlated with age, DR staging and retinol binding protein but negatively with body mass index, systolic blood pressure, diastolic blood pressure, fasting plasma glucose, and neutrophil count (all *P* < 0.05). There was no association between the serum magnesium levels and urea, uric acid, ischemia-modified albumin, calcium, lymphocyte count, platelet count (Table 5).

**TABLE 5:** Clinical characteristics correlated with the serum magnesium levels.

Related variables	*R*	*P*-value
Age (years)	0.126	0.004*
DR staging	0.118	0.007*
BMI (kg/m^2^)	–0.123	0.005*
SBP (mmHg)	–0.105	0.017*
DBP (mmHg)	–0.102	0.020*
Urea (mmol/L)	–0.007	0.868
UA (μmol/L)	–0.007	0.873
IMA (U/mL)	0.011	0.796
RBP (μg/mL)	0.092	0.036*
Calcium (mmol/L)	0.019	0.664
FPG (mmol/L)	–0.140	0.001*
Neutrophil (◊10^9^/L)	–0.121	0.006*
Lymphocyte (◊10^9^/L)	0.014	0.751
Platelet (◊10^9^/L)	–0.078	0.077

DR, diabetic retinopathy; BMI, body mass index; SBP, systolic blood pressure; DBP, diastolic blood pressure; UA, uric acid; IMA, ischemia modified albumin; RBP, retinol binding protein; FPG, fasting plasma glucose.

*P < 0.05, significant difference.

## Discussion

Diabetes is a serious public health concern in China. In 2018, the estimated prevalence of diabetes mellitus in China was 12.4%. Approximately 36.7% of the Chinese adults with diabetes reported being aware of their condition, 32.9% reported receiving treatment, and 50.1% of those receiving treatment had fully controlled disease. Compared with the United States, diabetes awareness, treatment, and control rates are lower in China (12). In many of our patients, diabetes was identified in the ophthalmology clinic after patients presented at the clinic with vision problems. Additionally, several patients with diabetes cannot be evaluated for fundus conditions due to severe cataract at the first ophthalmology visit. Without timely and effective treatment, DME may lead to severe vision loss or even blindness, which will impose a heavy economic burden on families and society. Therefore, timely detection and treatment of DME are important (3).

DME is a complex pathological condition caused by many factors. Retinal thickening in the macular area due to exudate accumulation (extracellular edema) caused by blood-retinal barrier dysfunction is considered the main pathological mechanism of DME (13). Magnesium is one of the major elements required to maintain normal metabolism and ionic balance in ocular tissues (14). A study involving 3,100 patients with diabetes with normal serum magnesium found that the incidence of diabetic retinopathy decreased with the increase of serum magnesium concentration. In patients with normal serum magnesium levels, the serum magnesium levels were negatively associated with the risk of diabetic microvascular complications (15). In another study involving 2,222 patients with diabetes, the authors stratified their serum magnesium levels into quartiles (Q1–Q4) and found that the group with low serum magnesium, Q1 (≤ 0.85 mmol/L), and Q2 (0.85–0.90 mmol/L), had significantly higher incidence rates of DR (50.9 and 30.2%, respectively) than the groups with high serum magnesium, Q3 (0.90–0.96 mmol/L), and Q4 (≥ 0.90 mmol/L) (23.5 and 21%, respectively). Lower serum magnesium levels were reportedly related to an increased risk of developing DR (9). However, the relationship between the serum magnesium levels and DME in patients with DR was not discussed in any of these studies. Our results suggest that within the normal range, higher serum magnesium level is a protective factor against DME in patients with DR, especially for those in non-proliferative DR period, aged < 66 years, who use insulin to control blood glucose.

Magnesium may protect against DME through systemic and local mechanisms. The serum magnesium concentration in normal adults is 0.70–1.10 mmol/L. Approximately 20% of this magnesium is protein bound, 65% is ionized, and the rest is combined with various anions, such as phosphates and citrates (16). Hypomagnesemia occurs in 13.5–47.7% of the patients with type 2 diabetes (17). Insulin resistance could reduce the TRPM6 channel activity of the intestinal and renal tubular epithelium and reduce the absorption of magnesium in the intestinal and renal epithelium, resulting in low serum magnesium (18). Simultaneously, hypomagnesemia can aggravate insulin resistance, and both of them are mutually pathogenic factors (19). Hyperglycemia is a systemic risk factor for DME (3). Magnesium intake reduces oxidative stress responses and improves insulin and glucose metabolism (20, 21). Therefore, patients with DR having high serum magnesium levels may have a reduced occurrence of DME because magnesium improves blood glucose and insulin resistance. Increased oxidative stress levels in the context of low serum magnesium levels may also contribute to DME progression. Retinal nerve tissue is rich in polyunsaturated fatty acids and has the highest oxygen consumption among all tissues. During magnesium deficiency, insufficient antioxidant enzyme activity can damage membranes rich in lipid peroxidation by free radicals, consequently impairing retinal function (22).

Inflammation is another cause of DME. It has been reported that supplementation with MgO or Mg picolinate has the greatest impact on retinal function as it can improve lipid peroxidation and reduce the expression of pro-inflammatory cytokines, such as intercellular adhesion molecule and vascular endothelial growth factor, by preventing oxidative stress and lipid production (23). Magnesium can reduce retinal oxidative stress and neuronal inflammation (22). However, deficiency in magnesium causes systemic and local inflammatory states that may predispose patients with DR to DME.

Moreover, magnesium has been shown to protect against heart failure in patients with type 2 diabetes. This protective effect is mediated, in part, by factors, such as the control of blood sugar by magnesium and the reduction of endothelial inflammation (24). Oral magnesium supplementation can improve vascular endothelial function and reduce vascular endothelial inflammation (25, 26). Destruction of retinal vascular endothelial cells is considered to be the initial cause of DME (13). Therefore, decreased serum magnesium levels may lead to the aggravation of retinal vascular endothelial inflammation, resulting in endothelial dysfunction and increased permeability, which may make patients with DR more prone to DME. In addition, hypomagnesemia causes an imbalance between vasoconstriction and dilation (24). Retinal vasospasm caused by magnesium deficiency leads to retinal ischemia and hypoxia, which further accelerate retinal damage (22).

Our data also suggest that insulin-treated patients with DR are at a higher risk of developing DME. Many people with type 2 diabetes require insulin therapy because of the gradual loss of islet beta cell function. Insulin use may also increase the risk of DR and DME in some patients (27). Among more than 300 patients treated with insulin, a 100% increased risk of DME was reported in those treated with insulin compared with those treated with oral medications (27). In addition, some studies have found that the macular thickness of patients with type 2 diabetes treated with insulin was higher than that of people in the control group (28). Possible mechanisms of action include the upregulation of vascular endothelial growth factor expression, vascular activity of insulin itself, sudden improvement in glycemic control, and further damage to the already-impaired blood-retinal barrier (4). Insulin can also disrupt the tight junctions of retinal pigment epithelial cells by disrupting the outer barrier (29). Hypomagnesemia among diabetic patients was associated with diabetic retinopathy (30), and a decreased level of serum magnesium was associated with an increase in macular thickness and ellipsoid zone disruption in DME (31). However, the correlation between serum magnesium, DME, and insulin use was not discussed. Interestingly, in our data, elevated serum magnesium reduced the risk of DME in patients with non-proliferative DR aged < 66 years who were treated with insulin, but not in other age groups or in patients who were not treated with insulin. This may be related to the fact that in some of our patients, diabetes was discovered only after the first diagnosis in ophthalmology because of vision problems, and no standardized endocrine therapy had been previously performed. Nevertheless, in multiple quadratic regression, our data elucidated that the risk of DME was reduced in the highest quartile of serum magnesium compared with the lowest quartile, suggesting that maintaining adequate magnesium levels may reduce the risk of DME in patients with DR. Our results highlight the need for interventional studies to assess whether magnesium supplementation reduces the risk of DME in patients with DR.

However, our study had some limitations. First, this was a retrospective study, and further prospective studies are needed to investigate the association between the serum magnesium levels and microvascular complications in patients with diabetes. In this retrospective study, we investigated the serum magnesium levels in patients with DR, and the assessment of the systemic magnesium status was incomplete. Second, some of the enrolled patients did not know their specific diabetes history. Third, selection bias may have existed. Most patients in this study were residents of Changshu, China; therefore, caution should be exercised when applying the conclusions of this study to other patient groups. Prospective multicenter studies are needed to validate magnesium as a predictor of DME risk.

## Conclusion

The higher the serum magnesium level, the lower the risk of DME in patients with DR. Furthermore, patients with DR who use insulin are more likely to develop DME. Long-term studies on oral magnesium supplements are needed to determine whether maintaining the serum magnesium levels within the physiological range can reduce the risk of DME in patients with DR.

## Data availability statement

The raw data supporting the conclusions of this article will be made available by the authors, without undue reservation.

## Ethics statement

The studies involving human participants were reviewed and approved by the Institutional Review Board of the Affiliated Changshu Hospital of Xuzhou Medical University (Changshu, China). The patients/participants provided their written informed consent to participate in this study.

## Author contributions

XX: conception and design, data analysis and interpretation, and manuscript writing. ZJ and TJ: data collection and collation. ZH and JY: data interpretation and final review of the manuscript. All authors revised and approved the submitted manuscript.

## Abbreviations

DME: diabetic macular edema
DR: diabetic retinopathy
OR: odds ratio.
